# New Integrated Strategy Emphasizing Infection Source Control to Curb *Schistosomiasis japonica* in a Marshland Area of Hubei Province, China: Findings from an Eight-Year Longitudinal Survey

**DOI:** 10.1371/journal.pone.0089779

**Published:** 2014-02-28

**Authors:** Yan-Yan Chen, Jian-Bing Liu, Xi-Bao Huang, Shun-Xiang Cai, Zheng-Ming Su, Rong Zhong, Li Zou, Xiao-Ping Miao

**Affiliations:** 1 Ministry of Education Key Lab of Environment and Health, Department of Epidemiology and Biostatistics, School of Public Health, Tongji Medical College, Huazhong University of Science and Technology, Wuhan, China; 2 Hubei Center for Disease Control and Prevention, Wuhan, China; Vanderbilt University, United States of America

## Abstract

**Background:**

Schistosomiasis remains a major public health problem in China. The major endemic foci are the lake and marshland regions of southern China, particularly the regions along the middle and lower reach of the Yangtze River in four provinces (Hubei, Hunan, Jiangxi, and Anhui). The purpose of our study is to assess the effect of a new integrated strategy emphasizing infection source control to curb schistosomiasis in marshland regions.

**Methods:**

In a longitudinal study, we implemented an integrated control strategy emphasizing infection source control in 16 villages from 2005 through 2012 in marshland regions of Hubei province. The interventions included removing cattle from snail-infested grasslands, providing farmers with mechanized farm equipment, improving sanitation by supplying tap water, building lavatories and latrines, praziquantel chemotherapy, controlling snails, and environmental modification.

**Results:**

Following the integrated control strategy designed to reduce the role of bovines and humans as sources of *Schistosoma japonicum* infection, the prevalence of human *S. japonicum* infection declined from 1.7% in 2005 to 0.4% in 2012 (*P*<0.001). Reductions were also observed in both sexes, across all age groups, and among high risk occupations. Moreover, the prevalence of bovine *S. japonicum* infection decreased from 11.7% in 2005 to 0.6% in 2012 (*P*<0.001). In addition, all the 16 villages achieved the national criteria of infection control in 2008.

**Conclusion:**

Our findings indicate that the integrated strategy was likely effective in controlling the transmission of *S. japonicum* in marshland regions in China.

## Introduction

Schistosomiasis is one of the most prevalent parasitic diseases in the world [1]. The global prevalence is currently estimated to be 207 million cases, with another 779 million people at risk of infection in 76 countries and territories [2], [3]. Documented evidence indicates that *Schistosoma japonicum* has been endemic for a long time in China [4]. *S. japonicum* eggs were identified in a male corpse dating back to the Western Han dynasty some 2100 years ago that was exhumed in Jianglin Hsien, Hubei Province in 1975 [5]. Schistosome eggs were also found in another female corpse buried about the same time in Hunan Province [6].

Schistosomiasis is mainly endemic today in lake and marshland areas (Hubei, Hunan, Jiangxi, Anhui, and Jiangsu provinces) and in hilly and mountainous regions (Sichuan and Yunnan provinces) in China [7]. The Chinese government has given high priority to the control of schistosomiasis since the founding of the People’s Republic of China in 1949. A number of special bodies were established to manage control activities from the national to the township level [4], [8], [9]. Schistosomiasis control strategies in China have gone through three stages since the 1950s. Prior to the 1980s, schistosomiasis controls primarily comprised waterway management and snail control. From 1980s to 2004, control measures were primarily synchronous chemotherapy for humans and domestic animals. Since 2005, integrated strategy emphasizing infection source control and mollusciciding were added to chemotherapy of humans. Significant achievements on schistosomiasis control have been attained through ongoing national control programs over the past 50 years [10]. The third nationwide schistosomiasis sampling survey indicated that the number of schistosomiasis patients decreased by 55.7%, from 1,638,103 cases in 1989 to 726,112 cases in 2004 [11].

However, each phase also has limitations. In the first stage, the endemic areas decreased immensely due to environmental modification, but severe environment pollution ensued because of the molluscicide widely used in endemic areas. Eliminating snails in lake and marshland habitats, in contrast to rivers and irrigation canals, has proved difficult [12]. In the second stage, human and bovine schistosomiasis were reduced to relatively low level temporarily, but re-infection occurred and continuous chemotherapy based schistosomiasis control could not be maintained [13]. At the beginning of the 21^st^ century, schistosomiasis was still of considerable economic and public health concern in China. The progress of schistosomiasis control has stalled since the termination of the World Bank Loan Project for schistosomiasis control at the end of 2001 [14], [15]. Data suggest that 110 counties had not yet reached the criteria for transmission control in 2003 [16]. The third national survey conducted in 2004 showed that the prevalence of *S. japonicum* infection in humans had not substantially changed in lakes, marshlands, and other areas of Southern China since 1995 [11].

In China, most of the current uncontrolled schistosomiasis endemic areas are concentrated in four provinces (Hubei, Hunan, Jiangxi, and Anhui) along the middle and lower reach of the Yangtze River. The interruption of transmission has proven particularly difficult to achieve, thus finding an effective and sustainable strategy for schitosomiasis control in the lake and marshland regions of China has become critical [16], [17], [18]. In 2004, the Chinese government directed much attention to the control of schistosomiasis, which was placed on the top priority list for the control of communicable diseases in China. Therefore, two control targets were established: to reduce human infection rates in all endemic counties to <5% by 2008, and then to <1% by 2015 [19]. A comprehensive strategy was developed to reduce the sources of infection (bovines and humans) in endemic regions of China [20].

Hubei province is located in the middle reaches of the Yangtze River. Following the effect of flood along the upper reaches of the Yangtze River during the annual monsoon season, the marshlands along the Yangtze River operate in a “winter–land, summer–water” cycle, and vast grass-covered marshlands emerge as floodwaters recede. These are ideal breeding sites for *Oncomelania hupensis* snails [21], [22]. *O. hupensis* is the unique intermediate host of *S. japonicum*, which has a key function during the transmission of schistosomiasis. Hubei province is a highly endemic area of schistosomiasis in China. Research has indicated a relatively high prevalence within the human population in Hubei province [18]. To achieve the two national control goals, an integrated control strategy aimed at reducing the roles of humans and cattle as the main sources of infection for snails was carried out in marshland areas of Hubei Province, China. The new strategy includes measures of improving water supply and sanitation, prohibiting the grazing of cattle in grasslands, captive feeding of livestock, and replacement of bovine with machines for farming [19].

In this paper, we examined this integrated control strategy which was carried out over an eight-year period in marshland areas of Hubei. Specifically we examined the effectiveness of this strategy in 16 study villages along the middle reaches of the Yangtze River, Hubei province from 2005 to 2012.

## Materials and Methods

### Study area

The study was conducted in the middle and lower reaches of the Yangtze River, Hubei province, China. In 2004, Hubei province had 5,499 schistosomiasis-endemic villages and 292,059 cases of chronic schistosomiasis; the prevalence of schistosomiasis in humans and bovines was 3.9% and 6.2%, respectively [23].

**Figure 1:**
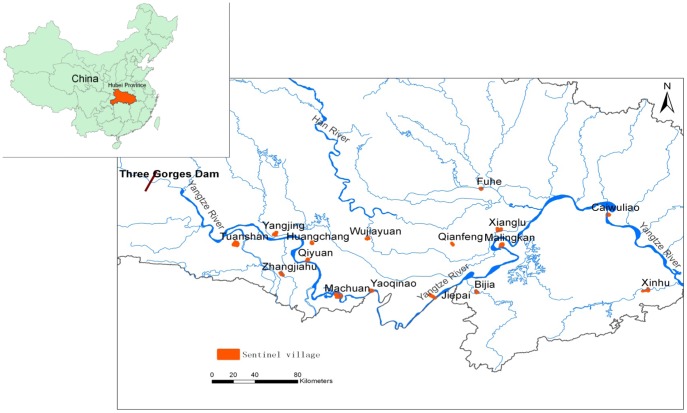
Location of the study villages in the mid-to-lower reaches of the Yangtze River, Hubei province, China.

We carried out a longitudinal survey in 16 villages from 16 counties in Hubei Province (Figure 1). The areas were selected through a three-stage random sampling procedure. The 16 counties were first randomly selected from 63 counties; then 16 towns were randomly selected from 16 counties; finally, 16 schistosomiasis-endemic villages were randomly selected from the 16 towns (each town selected a village). The 16 villages had approximately 23,835 people and 2,385 hm^2^ of agricultural acreage. Most of the residents were farmers whose principal activity was cultivation of rice and cotton. The residents were exposed to contaminated water when they performed their agricultural or daily activities (i.e., cultivating crops, catching fish, and washing clothes).

The participants met the following inclusion criteria: a) must have been a resident of the village for more than 12 months; b) should be more than six years old; c) should continuously reside in the village for the study period; d) has no serious diseases, such as malignant cancer.

### Integrated strategy emphasizing infection source control to curb schistosomiasis

The interventions were not only carried in study villages, but all the schistosomiasis-endemic villages in Hubei Province. The uptake of interventions in the study areas was no different than elsewhere in the province.

#### Interventions to control sources of S. japonicum infection

During the study period from 2005 to 2012, 575 cattle were replaced with small farm machines, representing 41.9% of the total cattle in the villages. The remaining bovines were not allowed to graze in marshlands containing known snail habitat and instead were confined to fences whenever possible. To reduce the potential of humans as a source of infection for snails, measures were implemented to attempt to reduce the transmission. Safe water was provided to 5,915 households by pipeline or well water supply, covering 97.6% of the total households. To deal with human feces, 5,696 home lavatories were constructed or repaired with three-cell septic tanks, representing 94.0% of the total households. Furthermore, fecal-matter containers were provided for fisherman to prevent them from excreting feces directly into river or lake freshwater.

#### Praziquantel chemotherapy

During the study period, all residents and bovines found positive for *S. japonicum* were treated with praziquantel (PZQ) (humans: 40 mg/kg; bovines: 25 mg/kg), in accord with World Health Organization recommendation [24]. For schistosomiasis japonica, cure rates of 70% to 90% have been recorded with these regiments. Those uncured also benefit because their egg counts fall to one fifth or less that of the pretreatment levels [25]. PZQ has also been used as a chemoprophylactic among high-risk groups, such as flood relief workers, tourists known to have been exposed recently and fishermen.

#### Comprehensive control of snail habitats

A comprehensive approach was employed to control snails by mollusciciding together with environmental modification. Mollusciciding using niclosamide was conducted after measuring snails twice a year in the 16 villages. Over eight years, 481.1 hm^2^ of snail habitats in the study villages were treated with molluscicides (niclosamide), and 54.6 hm^2^ were environmentally improved to control snails by ditch sclerosis, hardening river banks with concrete, sluice transformation, planting trees and constructing fish ponds.

### Outcome measurement

During the study period, all residents more than 6 years old in the 16 villages were examined using indirect hemagglutination assay (IHA) [26] in October and November annually, and IHA-positive individuals were subjected to the quantitative Kato–Katz thick smear technique (KK, three slides based on a single stool sample) [27]. *S. japonicum* egg counts were expressed in eggs/gram of stool (EPG). Residents that were positive for both IHA and KK were defined as infected, and the prevalence of *S. japonicum* in the residents of all 16 villages was determined in autumn.

During the same period, the infection of *S. japonicum* in all bovines (water buffaloes and cattle) in the 16 villages was examined annually using miracidial hatching test with fecal samples [28].

The snail survey was performed yearly along the river banks, ditches, and marshlands around the villages (April or May) through systematic sampling combined with environmental sampling (0.11 m^2^-sized frames, 20 m between frames) [29]. All snails within the square frames were collected and brought to the laboratory. The collected snails were then counted and crushed to examine microscopically for schistosome infections. The prevalence of infection of snails was calculated as the proportion of infected snails, and the density of infected snails as the number of infected snails/0.11 m^2^.

#### Infected snail area calculations

If an isolated infected snail spot has been found, then spread to each direction for 50 meters, that’s total of 10000 m^2^. If more than one infected snail spots have been found within 50 meters, then add the interval between each spot, and spread to each direction for 50 meters, than calculate the area. If more than one infected snail spots have been found exceed 50 meters, calculate the area by isolated infected snail spots.

### Ethical approval

The study was approved by the Ethics Review Committee of Hubei Provincial Center for Disease Control and Prevention, and the National Institute of Parasitic Diseases, Chinese Center for Disease Control and Prevention. Written informed consents were obtained from all adults and from parents or guardians of minors before participation in the study. The participants had the opportunity to withdraw from the study at any time.

The snail survey for each location was approved by Health Department of Hubei Province.

All animal work was approved by Ethics Review Committee of Animal Experiments, Hubei Provincial Center for Disease Control and Prevention. Animal cure and sacrifice were carried out by Animal Husbandry Bureau according to the guidance of the Institute for Laboratory Animal Research.

### Statistical analysis

Data were compiled in Microsoft Visual Foxpro database. Chi-square test or Fisher exact probability test were used to examine the proportions of differences. Spearman correlation was used to analyze the association between density and prevalence of snails. All *P* values were two-tailed, with a significant level at 0.05. All statistical analyses were carried out in SPSS 16.0.

## Results

### Baseline

The human *S. japonicum* prevalence in the 16 villages in 2005 was 1.7% (95% CI: 1.4–1.9) (N  =  12872) (Table 1).

**Table 1:** *S. japonicum* prevalence and intensity of infection in human in the 16 villages at baseline in 2005.

	Sub-group	No. of people examined	Prevalence ( 95% CI )
**All**		12872	1.65%(1.43,1.87)
**Sex**			
	Male	6655	2.04 % (1.70,2.38)
	Female	6218	1.22 % (0.95,1.50)
**Age**			
	6–9	667	0.30 % (–0.12,0.71)
	10–19	2481	0.85 % (0.49,1.21)
	20–29	920	1.09 % (0.42.1.76)
	30–39	2476	2.18 % (1.61,2.76)
	40–49	2912	2.16 % (1.64,2.69)
	50–59	1979	2.22 % (1.57,2.87)
	60-	1437	1.25 % (0.68,1.83)
**Occupation**			
	Farmer	9662	1.88 % (1.61,2.15)
	Fisherman	73	8.22 % (1.92,14.52)
	Student	2882	0.69 % (0.39,1.00)
	Other	255	1.57 % (0.04,3.09)

The number of males (N  =  6655) was greater than that of females (N  =  6218) in 2005. Infection prevalence was higher in males (2.0%; 95% CI: 1.7–2.4) than in females (1.2%; 95% CI: 1.0–1.5) (Table 1).

*S. japonicum* prevalence varied according to age. People between 50 to 59 years of age had the highest prevalence (2.2%; 95% CI: 1.6–2.9).

Majority of the residents were farmers (N  =  9662), followed by students (N  =  2882) (Table 1). The highest prevalence was found in the fisherman group (8.2%; 95% CI: 1.9–14.5) and the lowest was in the student group (0.7%; 95% CI: 0.4–1.0) (Table 1).

### Participant flow

Over the eight-year period, about 90% (ranged from 88.0% to 92.0%) of the qualified residents participated, and more than 95% of the IHA-positive individuals submitted a fecal sample for examination. PZQ treatment coverage was high, with 100% of those found infected successfully treated (Table 2).

**Table 2:** *S. japonicum* prevalence in human in the 16 villages from 2005 to 2012.

Year	No. of people qualified	No. of people tested	No. of people infected	Prevalence (95% CI)	Geometric mean EPG (95% CI)
2005	14514	12872	212	1.65 % (1.43, 1.87)	51.53(9.13,93,93)
2006	13032	11552	215	1.86% (1.61, 2.11)	16.50(10.10, 22.90)
2007	12471	11378	158	1.39 % (1.17, 1.60)	20.90(11.52,30.29)
2008	12725	11225	120	1.07 % (0.88, 1.26)	16.09(6.43,25.75)
2009	12027	10574	103	0.97% (0.79, 1.16)	19.98(3.09,36.87)
2010	11296	9941	86	0.86% (0.68, 1.05)	16.22(1.36,31.07)
2011	10536	9689	38	0.39 % (0.27, 0.52)	20.82(2.09,39.55)
2012	10699	9778	35	0.36% (0.24, 0.48)	18.34(3.67,33.01)

### *S. japonicum* infection in humans

#### Human prevalence

Human prevalence of *S. japonicum* infection declined from 2005 to 2012, showing a significant difference (χ^2^ = 204.2, *P*<0.001). The prevalence decreased from 1.7% in 2005 to 0.4% in 2012, showing a decline of 78.2% (*P*<0.001). The geometric mean intensity of infection in the positives decreased from 51.5 EPG in 2005 to 18.3 EPG in 2012, showing a decline of 64.4 % (Table 2).

#### Human prevalence by sex, age, and occupation over eight years

**Figure 2:**
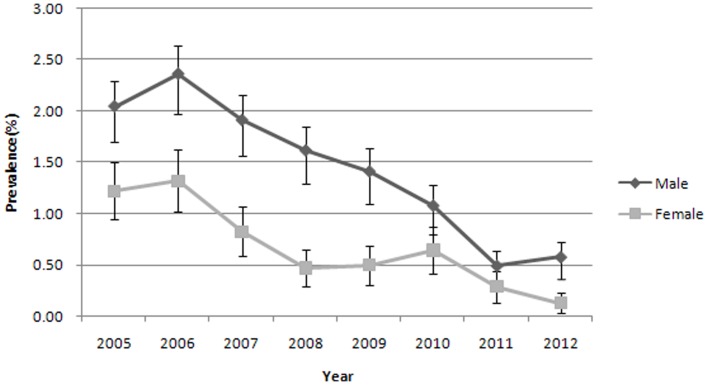
Human *S. japonicum* prevalence by sex and year (with 95% CI).

The human prevalence of *S. japonicum* infection by sex and year is shown in Figure 2. Prevalence was higher in males than in females over eight years. Significant difference between males and females was observed during the study period (*P*<0.05). The prevalence of *S. japonicum* in males decreased from 2.0% in 2005 to 0.6% in 2012, showing a decline of 71.6% (*P*<0.001). The prevalence of *S. japonicum* in females decreased from 1.2% in 2005 to 0.1% in 2012, showing a decline of 89.3% (*P*<0.001).

The human prevalence of *S. japonicum* infection by age and year is shown in Table 3. Human prevalence increased with age, and most infected residents were 30 years of age and older. Individuals younger than 30 years were infrequently infected, and the prevalence rate was low.

**Table 3:** Human *S. japonicum* prevalence by age in the 16 villages from 2005 to 2012.

Year	6–9		10–19		20–29		30–39		40–49		50–59		60-	
	No. tested	Prevalence %	No. tested	Prevalence %	No. tested	Prevalence %	No. tested	Prevalence %	No. tested	Prevalence %	No. tested	Prevalence %	No. tested	Prevalence %
2005	667	0.30 (–0.12, 0.71)	2481	0.85 (0.49,1.21)	920	1.09 (0.42,1.76)	2476	2.18 (1.61,2.76)	2912	2.16 (1.64,2.69)	1979	2.22 (1.57,2.87)	1437	1.25 (0.68,1.83)
2006	558	0.18 (–0.17,0.53)	1989	0.85 (0.45,1.26))	777	1.54 (0.68,2.41)	2136	2.81 (2.11,3.51)	2761	2.06 (1.53,2.59)	2091	2.10 (1.49,2.72)	1240	1.94 (1.17,2.70)
2007	502	0.20 (–0.19,0.59)	1887	0.26 (0.03,0.50)	734	0.41 (–0.05,0.87))	1912	1.46 (0.93,2.00)	2677	1.94 (1.42,2.47)	2231	2.06 (1.47,2.65)	1435	1.60 (0.95,2.25)
2008	357	0.00	1863	0.05 (–0.05,0.16)	838	0.48 (0.01,0.94)	1754	1.14 (0.64,1.64)	2712	1.62 (1.15,2.10)	2292	1.44 (0.95,1.93)	1409	1.28 (0.69,1.86)
2009	324	0.00	1492	0.00	652	0.77 (0.10,1.44)	1397	1.00 (0.48,1.52)	2677	0.86 (0.51,1.21)	2471	1.46 (0.98,1.93)	1561	1.60 (0.98,2.22)
2010	269	0.00	1105	0.00	577	0.17 (–0.17,0.51))	1119	0.80 (0.28,1.33)	2933	0.61 (0.33,0.90)	2284	1.31 (0.85,1.78)	1654	1.69 (1.07,2.31)
2011	239	0.00	881	0.00	840	0.12 (–0.11,0.35)	1184	0.25 (–0.03,0.54)	2908	0.34 (0.13,0.56)	2148	0.70 (0.35,1.05)	1489	0.60 (0.21,1.00)
2012	217	0.00	759	0.00	931	0.00	998	0.30 (–0.04,0.64)	2686	0.56 (0.28,0.84)	2270	0.44 (0.17,0.71)	1917	0.37 (0.10,0.64)

**Figure 3:**
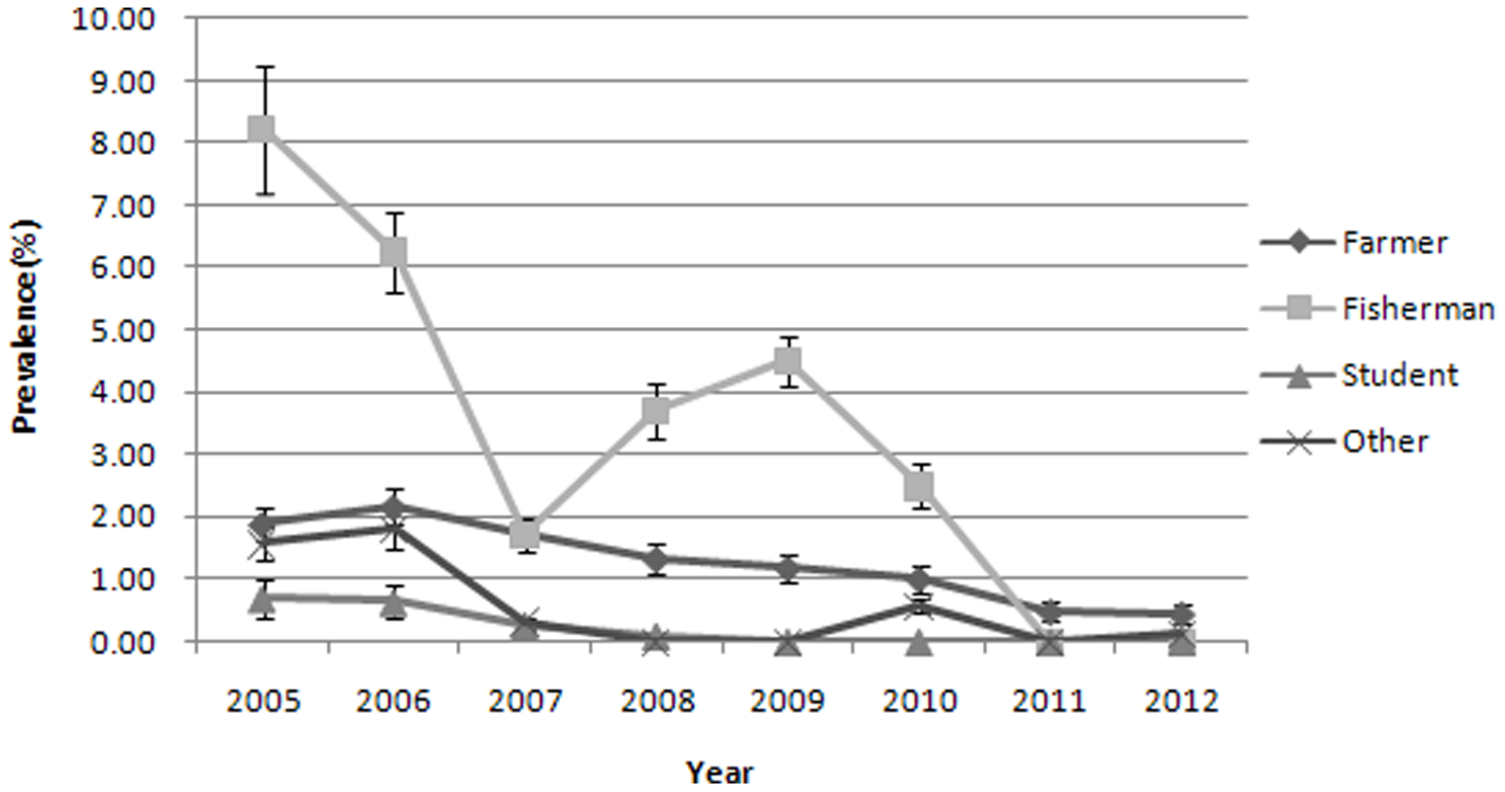
Human *S. japonicum* prevalence by occupation and year (with 95% CI).

The human prevalence of *S. japonicum* infection by occupation and year is shown in Figure 3 and Table 4. The majority of infected residents were fishermen and farmers, followed by students over eight years. *S. japonicum* prevalence decreased over the years. The prevalence in farmers decreased from 1.88% in 2005 to 0.4% in 2012, showing a decline of 77.1% (*P*<0.001). The prevalence in fishermen decreased from 8.2% in 2005 to 0% in 2012, showing a decline by 100% (*P* = 0.001).

**Table 4:** Human *S. japonicum* prevalence by occupation in the 16 villages from 2005 to 2012.

Year	Farmer			Fisherman			Student			Other		
	No. tested	No. infected	Prevalence %	No. tested	No. infected	Prevalence %	No. tested	No. infected	Prevalence %	No. tested	No. infected	Prevalence %
2005	9662	182	1.88	73	6	8.22	2882	20	0.69	255	4	1.57
2006	8932	193	2.16	48	3	6.25	2350	15	0.64	222	4	1.80
2007	8780	150	1.71	58	1	1.72	2229	6	0.27	311	1	0.32
2008	8692	115	1.32	81	3	3.70	2108	2	0.09	344	0	0.00
2009	8416	99	1.18	89	4	4.49	1784	0	0.00	285	0	0.00
2010	8221	82	1.00	80	2	2.50	1296	0	0.00	344	2	0.58
2011	8104	38	0.47	76	0	0.00	1141	0	0.00	368	0	0.00
2012	7912	34	0.43	90	0	0.00	903	0	0.00	873	1	0.11

### *S. japonicum* infection in bovines

*S. japonicum* prevalence in bovines is shown in Table 5. Significant difference (χ^2^  = 298.8,*P* <0.001) in prevalence was observed from 2005 to 2012. The prevalence decreased from 11.7% (95% CI: 9.5–13.9) in 2005 to 0.6% (95% CI: 0.0–1.1) in 2012, showing a decline of 95.0% (*P*<0.001).

**Table 5:** *S. japonicum* prevalence in bovines in the 16 villages from 2005 to 2012.

Year	No. tested	No. infected	Prevalence (95% CI)
2005	821	96	11.69% (9.49, 13.89)
2006	771	93	12.06% (9.76, 14.36)
2007	834	43	5.16% (3.66, 6.66)
2008	990	24	2.42% (1.47, 3.38)
2009	1162	23	1.97% (1.18, 2.78)
2010	780	11	1.41% (0.58, 2.24)
2011	725	6	0.83% (0.17, 1.49)
2012	693	4	0.58 % (0.01, 1.14)

### *S. japonicum* infection in snails

The infected snail habitat decreased from 58.95 hm^2^ in 2005 to 0 hm^2^ in 2012, showing a decline of 100%. The snail infection decreased from 0.3% (95% CI: 0.2–0.3) in 2005 to 0% in 2012, showing a decline of 100% (Table 6).

**Table 6:** *S. japonicum* prevalence in snail in the 16 villages from 2005 to 2012.

Year	No. snails dissected	Density of living snails(No./0.11 m^2^)	No. infected snails	Infected snail habitat (hm^2^)	Density of infected snails (No./0.11 m^2^)	Prevalence (95% CI)
2005	46978	0.85	138	58.95	0.0025	0.29% (0.24, 0.34)
2006	35328	0.67	60	64.72	0.0011	0.17% (0.13, 0.21)
2007	22386	0.44	38	35.78	0.0008	0.17% (0.12, 0.22)
2008	14601	0.28	33	27.71	0.0006	0.23% (0.15, 0.30)
2009	15824	0.29	22	18.08	0.0004	0.14% (0.08, 0.20)
2010	16914	0.32	10	4.07	0.0002	0.06% (0.02, 0.10)
2011	13927	0.19	3	1.13	0.00004	0.02% (0.00, 0.05)
2012	15010	0.20	0	0	0	0

The density of living snails decreased from 0.9 No./0.11 m^2^ in 2005 to 0.2 No./0.11 m^2^ in 2012, showing a decline of 76.5%. The density of infected snails decreased from 0.003 No./0.11 m^2^ in 2005 to 0 No./0.11 m^2^ in 2012, showing a decline of 100%. The prevalence of *S. japonicum* infection in the snails decreased from 0.3% in 2005 to 0% in 2012, showing a decline of 100% (Table 6).

Significant correlations were observed between the density of infected snails and the density of living snails (r  =  0.881, *P* =  0.004).

## Discussion

Investigations showed that more than 80% of *S. japonicum* patients in China were distributed in the lake and marshland areas of Hubei, Hunan, Jiangxi, Anhui, and Jiangsu provinces [30]. In 2005, 291,111 cases of *S. japonicum* patients, 14,816 heads of infected bovines, and 76,435 hm^2^ of areas infested with *Oncomelania* snails were reported in Hubei province, accounting for the highest numbers in China [31]. Therefore, schistosomiasis control in Hubei province is especially important.

To achieve the compliance requirements of the national prevention plan, the Hubei Provincial government formulated a document called “Mid- and long-term plan on prevention and control of schistosomiasis in Hubei province (2005–2015)”. Hubei province has conducted a series of comprehensive prevention and control measures based on blocking the transmission of *S. japonicum* from cattle/buffalos and humans to snails since 2005 [20]. We selected 16 villages and used longitudinal observation for eight years to evaluate *S. japonicum* transmission following comprehensive prevention and control measures.

The prevalence of human *S. japonicum* infection declined considerably in all surveyed villages over the course of the study. In 2008, the prevalence of human *S. japonicum* infection decreased to below 5% and no acute schistosomiasis case was reported. The national criterion of infection control was achieved. In the following years, the prevalence continued to decline. The integrated control strategy had an active and important function in the decrease in human prevalence observed in the study villages. The decrease in human prevalence attributable to human fecal contamination of the environment was effectively controlled by sanitary latrine construction. Annual PZQ treatment, which on ethical grounds had to be administered to all infected individuals, may also have helped.

In the study, we found that infection prevalence was higher in males than females in the study villages. This finding may be related to the fact that males often take part in agricultural labor and have more exposure to infested water. To a far lesser extent, females are exposed while bathing or washing clothes. Male-dominated exposure has been reported elsewhere in China [32]. In Jiangxi Province, China, most of the reported contact for males occurred while washing or fishing [32]. The result showed that the infected population was mainly concentrated in ages 30 to 60. Infection prevalence was low in children, which may due to the health education carried in schools every year. Health education plays an important role on schistosomiasis control as part of the integrated strategy[33]. Our previous research had found that heath education was effective on reducing the prevalence of *S. japonicum* infection in human especially students[34]. Moreover, some prevalent cases may be persons with long-lived *S. japonicum* [35]. Thus, most human exposures to schistosomiasis in a typical Chinese lake and marshland region are occupationally driven.

A number of reports have shown that bovines are the major transmission sources for human schistosomiasis in the lakes and marshland areas of Southern China, and that interventions targeting bovines can reduce the incidence of human infection [36], [37]. Over the study period, bovine prevalence rates decreased in 16 villages, possibly due to measures including providing farmers with machines instead of bovines and prohibiting bovines in regions with snails. These measures have been shown to be effective elsewhere [19]. However, some challenges exist for the implementation of these measures in some rural areas of Hubei Province. Not all farms were suitable for tractor farming and bovines also bring additional economic benefits to villages beyond their use in farming [38].

The densities and rates of *S. japonicum* infection in snails were consistent with the prevalence of human *S. japonicum* infection. Our findings suggest that environmental management is very effective for snail control, and could result in a persistent reduction in the density of living snails. However, environmental management is limited by the floods. Major flooding of the lakes and marshland areas downstream of the Three Gorges Dam (TGD) can drown adult snails, resulting in decreased transmission [7].

During the past years, the Hubei Provincial government placed a high priority on the control of schistosomiasis and carried out numerous control programs [39], [40], [41]. Our research suggested that the integrated control strategy implemented in our study area was effective. Earlier studies showed that the chemotherapy-based approach could not perennially reduce the prevalence of *S. japonicum* [42], [43]. Given the decreasing drug efficacy after widespread and repeated use of PZQ, chemotherapy alone can only control the infection rates at a low level; it cannot eliminate re-infections, and therefore, cannot interrupt the transmission of schistosomiasis. However, the key for integrated control strategy is to manage feces of humans and animals, preventing the eggs from entering the water so the snails will not be infected, reducing human and animal re-infected [43].

However, some challenges still remain for schistosomiasis control in Hubei province, China. Schistosomiasis endemic may be infected by large-scale hydro-projects [7], [44], [45]. The ecology of central China will change substantially in ways that could affect the distribution and transmission of schistosomiasis due to the TGD [25], [46]. It is a giant impoundment across the Yangtze River, which was close to a height of 135 m by 2003 and reached its full height of 175 m by 2009. TGD has been predicted to alter water and sand distributions downstream, which will have a significant effect on ecological systems; these include the canals of Hubei and Dongting and Poyang lakes, where *S. japonicum* transmission is generally projected to increase, although decreased transmission is projected for other locations [7], [22], [47]. Specifically, TGD is anticipated to result in large changes to the flow, depth, and sedimentation load of the Yangtze; thus, the distribution and number of schistosome-infected *Oncomelania* snails will be altered, increasing transmission of schistosomiasis in some areas and its re-introduction into places where the infection is currently under control [7], [21], [48], [49]. Gray and colleagues believe that TGD has no immediate effect on schistosome transmission, and that the predicted changes may take longer to occur [50]. Continued surveillance should be undertaken to monitor the future ecological effects of the dam in Hubei province.

Large population movements taking place in southern China have exacerbated the schistosomiasis problem [47]. The downturn in the world economy in 2007 has also induced further population movements as businesses in urban centers close and employment opportunities recede. The foregoing is likely to affect schistosomiasis control efforts negatively, with millions of residents returning from cities to their home villages in areas of endemicity to seek employment.

In our research, the densities and rates of infected snails were both at a low level, possibly caused by the low sensitivity of the microscopy-based method; identifying snails that were infected early through this microscopy method is very difficult [51]. To assess the long-term effectiveness of the integrated strategy and to maintain the success of this strategy, using these new molecular tools to monitor snails in the future may be necessary. Molecular tools, such as polymerase chain reaction and loop-mediated isothermal amplification, can detect potential infection in snails with early sporocysts, and show promise for monitoring early infection rate in snails [51].

Schistosomiasis control is a systematic project involving sectors of water conservancy, agriculture, forestry, health, and others. External factors of equal importance, such as political, economic, cultural and social, should not be ignored [52]. With the development of the social economy, the living standards of residents in the endemic areas will be improved continually, so control of schistosomiasis will become easier. This study shows that an integrated strategy, predominantly involving the elimination of infection sources in the environment and snail control, combined with treatment of infected humans is achievable and effective[43]. Therefore, a multi-component, integrated control program will be continuously required to combat the spread of schistosomiasis [53] and to achieve the national criteria of transmission control to reduce the prevalence of *S. japonicum* to less than 1% in Hubei Province.

Our study had somewhat potential limitations. The interventions were carried province-widely, thus there are no available uncontrolled villages for comparison. Our study data could represent the basic schistosomiasis endemic of the province as a whole because of random selection and 8-years longitudinal survey were carried. Though there may have bias when selected 16 villages from 5502 villages in the province with random selection.

We did not investigate the status of *S. japonicum* infection in people who withdraw from the study. Thus, we did not know the differential dropout of infected persons. The study populations shrunk over the course of the study, though most of the dropouts were below 30 years and were not the high risk persons. Moreover, we tested about 90% of the eligible persons each year. So we consider that the limitations of study have little effect on the results.

## Conclusions

An integrated control strategy aimed at controlling the roles of bovines and humans as sources of *S. japonicum* infection is suggested to be likely effective in controlling the transmission of *S. japonicum* in marshland regions of China.
